# Differences in renal stone treatment and outcomes for patients treated either with or without the support of a ureteral access sheath: The Clinical Research Office of the Endourological Society Ureteroscopy Global Study

**DOI:** 10.1007/s00345-015-1582-8

**Published:** 2015-05-14

**Authors:** Olivier Traxer, Gunnar Wendt-Nordahl, Hiren Sodha, Jens Rassweiler, Shimon Meretyk, Ahmet Tefekli, Fernando Coz, Jean J. de la Rosette

**Affiliations:** Department of Urology, University Pierre et Marie Curie, Paris, France; Department of Urology, Sindelfingen-Böblingen Medical Center, Sindelfingen, Germany; Department of Urology, RG Stone Urology and Laparoscopy Hospital, Andheri, Mumbai, India; Department of Urology, SLK-Klinikum am Gesundbrunnen, Heilbronn, Germany; Department of Urology, Rambam Medical Center, Faculty of Medicine, Technion-Israel Institute of Technology, Haifa, Israel; Department of Urology, School of Medicine, Bahcesehir University, Istanbul, Turkey; Department of Urology, Hospital Militar, Universidad de Los Andes, Santiago, Chile; Department of Urology, AMC University Hospital, Meibergdreef 9, 1105 AZ Amsterdam, The Netherlands

**Keywords:** Ureteroscopy, Renal stones, Ureteral access sheath treatment outcome, Complications

## Abstract

**Purpose:**

To describe the differences in the treatment and the outcomes of renal stones treated with flexible ureteroscopy (URS) either with or without the support of a ureteral access sheath (UAS).

**Methods:**

The Clinical Research Office of the Endourological Society URS Global Study involved the collection of prospective data from consecutive patients treated with URS at centers around the world over a 1-year period. Baseline characteristics, stone location, treatment details, postoperative outcomes and complications were recorded. Inverse-probability-weighted regression adjustment (IPWRA) analyses were conducted on outcome from patients treated with or without the use of a UAS to determine the impact on stone-free rates (SFRs).

**Results:**

Of 2239 patients treated with flexible URS, 1494 (67 %) patients were treated with the use of a UAS and 745 (33 %) without a UAS. The IPWRA analyses conducted on 1827 patients with complete data and based on treatment and outcome models showed that if URS procedures were performed without the use of an UAS, the average stone-free rate would be 0.504 compared with 0.753 with a UAS. This average treatment effect of 0.248 was not significant (*P* = 0.604). Using IPWRA analysis on only the treated population in the estimations revealed no significant difference between using and not using a UAS (31 %; ATET: 0.311; *P* = 0.523).

**Conclusions:**

The study showed no difference in SFR when a UAS was used or not. Whereas UAS did not increase the risk of ureteral damage or bleeding, postoperative infectious complications were reduced.

## Introduction

The ureteral access sheath (UAS) has been developed on the same concept as AMPLATZ sheath for percutaneous nephrolithotomy, i.e., to allow direct access to the kidney and to decrease the intra-renal pressure during upper-tract endourological procedures. It has become increasingly popular as it offers a number of potential advantages including facilitation of access to the renal collecting systems, multiple entry and reentry, decreased intra-renal pressure, and improved drainage around the scope [1–5]. The UAS has the added advantage of allowing passive elimination of small stone fragments generated during laser fragmentation with the exit of irrigation fluid. Nevertheless, the use of the UAS can itself cause damage to the ureter by over distension which compromises ureteral blood flow, resulting in ureteral ischemia [6] or direct damage to the ureter during the insertion of the UAS [7].

The CROES initiated the Ureteroscopy (URS) Global Study to establish a prospective global database to examine the worldwide use of URS and determine factors affecting outcome. In the currently presented study, inverse-probability-weighted regression adjustment (IPWRA) is used to describe differences in stone-free rate (SFR) of patients who underwent URS treatment for their renal stones, with or without the use of a UAS.

## Patients and methods

### Study population

The URS Global Study is a prospective, observational, international, multicenter study with data collected on consecutive patients treated with URS over a 1-year period at each participating center. The overall study period was from January 2010 until October 2012. Centers were asked to treat patients according to their local protocols. Institutional Research Board (IRB) or Institutional Ethics Committee approval was obtained by all participating centers before the start of the study. If IRB was not needed, the centers followed the protocol according the rules of Good Clinical Practice.

Patients who were eligible for inclusion in the present analysis were those who were candidates for flexible URS for renal stones as a primary treatment or after failure of a previous treatment and aged ≥18 years. No specific exclusion criteria were applied. Details of treatment including secondary treatment and patient follow-up have been described previously [8].

### Assessment

Data were encrypted and collected electronically through a web-based Web site: www.croesoffice.org and held in a central database at the CROES office. Data included patient epidemiological characteristics, calculus specification, type of treatment, and postoperative outcomes and complications. Stone burden was calculated as the sum of all stone sizes (length × width × 0.25 × 3.14159). The classification of a patient being stone free (SFR) was based on the absence of stones or fragments >1 mm. Operating time was defined as the time from the insertion of the endoscope until the insertion of a bladder catheter. Treatment failure was defined as stone still in situ, remaining stone fragments >1 mm, and failed access. Treatment success was defined as a patient free of stones >1 mm.

### Confounders

Preoperative possible confounders were body mass index (BMI), age, gender, stone location, stone size, stone burden, American Society of Anesthesiologists (ASA) score, preoperative stent placement, anticoagulant and antibiotics use, comorbidities such as diabetes, cardiovascular disease (CVD), prednisone use, Crohn’s disease, renal congenital abnormalities, a solitary kidney, case volume and academic status of the operating center. Intra-operative possible confounders were type or URS, type of fragmentation device, operation time, intra-operative complications and a postprocedural stent placement. Postoperative possible confounders were the method of evaluation, the length of hospital stay, retreatment, readmission and postoperative complications.

### Statistical analysis

Inverse-probability-weighted regression adjustment (IPWRA) analyses were performed to determine whether there was a difference in SFR between using and not using of a UAS, independent from well-known possible confounders. Inversed probability weighting (IPW) is a method in which each observation is weighted by the propensity score of individual observations. If a subject had a higher probability of being in a group (using UAS or not), it was considered as overrepresented and therefore was given a lower weight. Alternatively, if the patient had a smaller probability of being in the group, it was considered as underrepresented and was given a higher weight. Generally, in an observational study, the weighting adjustment removes sampling bias [9]. Subsequently, IPWRA is a regression analysis in which preoperative characteristics-based inverse probability weights are used in a regression model describing the relationship of interest.

## Results

### Patient characteristics

A total of 2239 patients with renal stones were treated with flexible URS. Of these 1494 (67 %) patients were treated with the use of a UAS and 745 (33 %) without a UAS. Baseline characteristics are shown in Table 1. Overall, subjects treated with an UAS have higher preoperative ASA scores and larger stones.

**Table 1:** Preoperative characteristics of patients with renal stones treated with a flexible ureteroscopy (URS) with or without the assistance of a ureteral access sheath (UAS)

Parameter	Patients treated with UAS (*n* = 1494)	Patients treated without UAS (*n* = 745)	Total population (*n* = 2239)	Group difference statistic
Age (years), mean (SD)	51.2 (14.98) (*n* = 1494)	50.2 (14.95) (*n* = 745)	50.9 (14.98) (*n* = 2239)	NS
Gender				
Male, *n* (%)	901 (60.3)	462 (62.0)	1363 (60.9)	
Female, *n* (%)	593 (39.7) (*n* = 1494)	283 (38.0) (*n* = 745)	876 (39.1) (*n* = 2239)	NS
BMI, mean (SD)	27.8 (7.4) (*n* = 1398)	27.4 (5.8) (*n* = 606)	27.7 (6.93) (*n* = 2004)	NS
Comorbidity, *n* (%)				
DM	192 (12.9) (*n* = 1483)	100 (13.7) (*n* = 729)	292 (13.2) (*n* = 2212)	NS
CVD	503 (34.0) (*n* = 1479)	193 (26.5) (*n* = 729)	696 (31.5) (*n* = 2208)	NS
Prednisone	28 (1.9) (*n* = 1479)	6 (0.8) (*n* = 730)	34 (1.5) (*n* = 2208)	NS
Crohn’s disease	15 (1.0) (*n* = 1479)	13 (1.8) (*n* = 731)	28 (1.3) (*n* = 2210)	NS
Anticoagulation	153 (10.4) (*n* = 1475)	57 (7.8) (*n* = 729)	210 (9.5) (*n* = 2204)	NS
Antibiotics	1312 (88.8) (*n* = 1478)	662 (91.7) (*n* = 722)	1974 (89.7) (*N* = 2200)	NS
Renal stone location, *n* (%)				
Upper pole	115 (7.9)	63 (8.6)	178 (8.1)	NS
Mid pole	118 (8.1)	78 (10.6)	196 (9.0)	
Lower pole	505 (34.9)	271 (36.8)	776 (35.5)	
Renal pelvis	253 (17.5)	130 (17.7)	383 (17.5)	
Multiple	458 (31.6) (*n* = 1449)	194 (26.4) (*n* = 722)	652 (29.8) (*n* = 2082)	
Stone size, *n* (%)				
<10 mm	720 (51.9)	458 (65.8)	1178 (56.6)	*P* < 0.01
≥10 mm	666 (48.1) (*n* = 1386)	238 (34.2) (*n* = 696)	904 (43.4) (*n* = 2082)	
Stone burden, mean (SD)^a^	108.3 (114.4) (*n* = 1287)	99.2 (100.5) (*n* = 658)	102.2 (109.6) (*n* = 1945)	NS
Previous calculus treatment, *n* (%)	765 (51.8) (*n* = 1476)	316 (42.8) (*n* = 739)	1081 (48.8) (*n* = 2215)	NS
PCNL	206 (14.0) (*n* = 1476)	58 (7.8) (*n* = 738)	264 (11.9) (*n* = 2218)	
SWL	408 (27.7) (*n* = 1473)	163 (22.1) (*n* = 738)	571 (25.8) (*n* = 2211)	
Ureterolithotomy	11 (0.7) (*n* = 1479)	10 (1.3) (*n* = 741)	21 (0.9) (*n* = 2220)	
URS	384 (26.0) (*n* = 1475)	168 (22.6) (*n* = 742)	552 (24.9) (*n* = 2217)	
Pyelolithomy	25 (1.7) (*n* = 1484)	10 (1.3) (*n* = 742)	35 (1.6) (*n* = 2226)	
UPJ pyeloplasty	12 (0.8) (*n* = 1479)	4 (0.5) (*n* = 740)	16 (0.7) (*N* = 2222)	
Solitary kidney	46 (3.1) (*n* = 1482)	19 (2.6) (*n* = 740)	65 (2.9) (*n* = 2222)	NS
Renal congenital abnormality, *n* (%)	105 (7.0)	40 (6.4)	145 (6.5)	NS
Horse shoe	18 (1.2)	4 (0.5)	22 (1.0)	
Ectopic	4 (0.3)	4 (0.5)	8 (0.4)	
Malrotation	4 (0.3) (*n* = 1490)	3 (0.4) (*n* = 741)	7 (0.3) (*n* = 2231)	
Preoperative stent, *n* (%)	533 (35.8)	284 (38.4)	816 (36.7)	NS
Double J	511 (95.9)	278 (98.2)	789 (96.7)	
Single J	8 (1.5)	–	8 (1.0)	
Ureteric catheter	3 (0.6) (*n* = 1487)	2 (0.7) (*n* = 739)	5 (0.6) (*n* = 1432)	
ASA score, *n* (%)				
I	512 (36.4)	267 (41.4)	779 (38.0)	*P* ≤ 0.01
II	649 (46.2)	266 (41.2)	915 (44.6)	
III	228 (16.2)	105 (16.3)	333 (16.2)	
IV	16 (1.1) (*n* = 1405)	7 (1.1) (*n* = 645)	23 (1.1) (*n* = 2050)	
Case volume, *n* (%)				
Low-volume center	314 (21.0)	149 (20.0)	463 (20.7)	NS
High-volume center	1180 (79.0) (*n* = 1487)	596 (80.0) (*n* = 745)	1776 (79.3) (*n* = 2082)	

Sample sizes for which data were available are shown in parenthesis

Group differences were tested with a *t* test for continuous variables, a Chi-square test for dichotomous and an ANOVA for categorical variables

*BMI* body mass index, *DM* diabetes mellitus, *CVD* cardiovascular disease, *PCNL* percutaneous nephrolithotomy, *SWL* extracorporeal shockwave lithotripsy, *URS* ureteroscopy, *UPJ* ureteropelvic junction, *ASA* American Society of Anesthesiologists

^a^Stone burden was calculated as the sum of all stone sizes (length × width × 0.25 × 3.14159)

Additionally, the UAS was most often used in North America (71.9 %), and South America (76.1 %), compared with Asia (67.3 %), Western Europe (66.1 %) and Eastern Europe (58.3 %). Systematic use (≥80 % of the procedures) of a UAS occurred in 39 centers (47 %), and never in 14 centers (17 %).

### Operating parameters

Intra-operative characteristics are shown in Table 2. More patients were treated flexible URS alone in the UAS group and more patients with flexible plus semirigid URS in the non-UAS group. Also, size of the stones is larger in the UAS group, complication rates differ between the groups, and operating time is longer in the UAS group. Laser fragmentation was the most common form of stone disruption used.

**Table 2:** Intra-operative characteristics of patients with renal stones treated with a flexible ureteroscopy (URS) with or without the assistance of a ureteral access sheath (UAS)

Parameter	Patients treated with UAS (*n* = 1494)	Patients treated without UAS (*n* = 745)	Total population (*n* = 2239)	Group difference statistic
Type of URS, *n* (%)				*P* < 0.01
Flexible	973 (65.1)	416 (55.8)	1389 (62.0)	
Flexible + semirigid	521 (34.9) (*n* = 1494)	329 (44.2) (*n* = 745)	552 (38.0) (*n* = 2239)	
Fragmentation device, *n* (%)				
Laser	1304 (87.9)	609 (82.2)	1913 (86.0)	NS
Other	13 (0.8)	16 (2.1)	29 (1.3)	
None	167 (11.3) (*n* = 1484)	116 (15.7) (*n* = 741)	283 (12.7) (*n* = 2225)	
Operating time (mins, mean [SD])	80.0 (44.2) (*n* = 1414)	64.7 (37.3) (*n* = 722)	74.8 (42.6) (*n* = 2136)	*P* ≤ 0.01
Stone size sub-categories, *n* (%)				*P* ≤ 0.01
<10 mm	720 (51.9)	458 (65.8)	1178 (56.6)	
≥10 mm	666 (48.1) (*n* = 1386)	238 (34.2) (*n* = 696)	904 (43.4) (*n* = 2082)	
Intra-operative complication, *n* (%)				NS
Uneventful	1402 (94.1)	706 (94.9)	2108 (94.4)	
Failed	12 (0.8)	13 (1.7)	25 (1.1)	
Bleeding	31 (2.1)	9 (1.2)	40 (1.8)	
Perforation	17 (1.1)	9 (1.2)	26 (1.2)	
Other	5 (1.0)	10 (1.3)	30 (1.3)	
Converted	3 (0.2)	1 (0.1)	4 (1.2)	
Avulsion	– (*n* = 1490)	− (*n* = 744)	– (*n* = 2234)	
Postoperative stent, *n* (%)	1352 (90.6) (*n* = 1493)	611 (82.0) (*n* = 745)	1963 (87.7) (*n* = 2238)	NS

Sample sizes for which data were available are shown in parenthesis

Group differences were tested with a *t* test for continuous variables, a Chi-square test for dichotomous and an ANOVA for categorical variables

### Postoperative outcome and complications are shown in Table 3

Stone-free rates were 73.3 versus 59.3 % for the smaller stones and 81.5 versus 84.9 % for larger stones with and without a UAS, respectively; SFRs were lower with the use of a UAS (73.9 vs. 82.8 %). Higher SFR is most outspoken in cases with multiple stone locations. Also, hospital stay is longer in cases treated with an UAS.

**Table 3:** Postoperative characteristics of patients with renal stones treated with a flexible ureteroscopy (URS) with or without the assistance of a ureteral access sheath (UAS)

Parameter	Patients treated with access sheath (*n* = 1494)	Patients treated without access sheath (*n* = 745)	Total population (*n* = 2239)	
Stone-free rate, *n* (%)	1051 (73.9) (*n* = 1422)	602 (82.8) (*n* = 727)	1653 (78.0) (*n* = 2149)	*P* < 0.01
SFR by location sub-categories, *n* (%):				
Upper pole	84 (76.4)	52 (83.9)	136 (79.0)	NS
Mid pole	97 (84.3)	69 (89.6)	166 (86.5)	NS
Lower pole	386 (79.8)	222 (82.8)	608 (80.9)	NS
Renal pelvis	188 (78.3)	102 (82.9)	290 (79.9)	NS
Multiple	263 (61.3) (*n* = 1018)	148 (79.0) (*n* = 593)	411 (66.7) (*n* = 1611)	*P* < 0.01
SFR by stone size sub-categories, *n* (%)				
<10 mm	118(73.3)	269 (59.3)	387 (62.9)	NS
≥10 mm	712 (81.5) (*n* = 830)	444 (84.9) (*n* = 713)	1156 (82.7) (*n* = 1543)	*P* ≤ 0.01
Method of evaluation, *n* (%)				NS
Ultrasound	572 (38.8)	331 (44.7)	903 (40.7)	
X-ray/KUB	724 (49.1)	285 (38.5)	1009 (45.5)	
CT	292 (19.8)	83 (11.2)	375 (16.9)	
IVU	81 (5.5)	20 (2.7)	101 (4.6)	
Retrograde pyelogram	20 (1.4)	66 (8.9)	86 (3.9)	
Intra-operative confirmation	246 (16.7)	99 (13.4)	345 (15.6)	
Other	16 (1.1)	4 (0.5)	20 (0.9)	
None	142 (9.6) (*n* = 1476)	29 (3.9) (*n* = 741)	171 (7.7) (*n* = 2217)	
Hospital stay (d), mean (SD)	1.47 (2.24) (*n* = 1481)	1.69 (2.92) (*n* = 738)	1.55 (2.48) (*n* = 2219)	*P* = 0.025
Retreatment, *n* (%)	142 (9.5) (*n* = 1494)	60 (8.1) (*n* = 743)	202 (9.0) (*n* = 2237)	NS
Postoperative complication, *n* (%)	102 (6.8) (*n* = 1494)	40 (5.4) (*n* = 745)	142 (6.8) (*n* = 2239)	NS
Complication according to stone location sub-categories, *n* (%):				NS
Upper pole	8 (7.1)	5 (12.5)	13 (8.6)	
Mid pole	12 (10.7)	4 (10.0)	16 (10.5)	
Lower pole	32 (28.6)	12 (30.0)	44 (28.9)	
Renal pelvis	22 (19.6)	10 (25.0)	32 (21.1)	
Multiple	38 (33.9) (*n* = 112)	9 (22.5) (*n* = 40)	47 (30.9) (*n* = 152)	
Complication according to stone size sub-categories:				NS
<10 mm	52 (48.6)	24 (60.0)	76 (51.7)	
≥10 mm	55 (51.4) (*n* = 107)	16 (40.0) (*n* = 40)	71 (48.3) (*n* = 147)	
Total postoperative complications*, n* (%)	115 (7.7)	40 (5.4)	155 (6.9)	*P* = 0.041
Bleeding	9 (5.6)	4 (8.7)	13 (5.9)	
Fever	46 (28.6)	18 (39.1)	64 (29.4)	
UTI	30 (18.6)	11 (23.9)	41 (18.8)	
Bladder cramps	13 (8.1)	5 (10.9)	18 (16.2)	
Lung embolism	1 (0.6)	–	1 (0.5)	
CVA	–	1 (2.2)	1 (0.5)	
Sepsis	7 (4.3)	7 (15.2)	14 (6.4)	
Acute abdomen	2 (1.2)	–	2 (0.9)	
Other	53 (32.9) (*n* = 1491)	11 (23.9) (*n* = 743)	64 (29.4) (*n* = 2234)	
Readmission <3 months *n* (%)	180 (13.1) (*n* = 1372)	63 (9.1) (*n* = 693)	243 (11.8) (*n* = 2065)	NS

Sample sizes for which data were available are shown in parenthesis

Group differences were tested with a *t* test for continuous variables, a Chi-square test for dichotomous and an ANOVA for categorical variables

*CT* computed tomography, *KUB* kidneys, ureters and bladder, *IVU* intravenous urography, *UTI* urinary tract infection, CVA cardiovascular accident

### Regression analysis

Of the total study population, only those who had complete information (*n* = 1827) were used in the regression analysis. The IPWRA analyses based on both treatment and outcome models are shown in Table 4. In this model, it was found that if none of the URS procedures were performed with the use of an UAS, the average stone-free rate would be 0.504, i.e., as much as 50 % of the population would be stone free. In addition, if all of the URS procedures were performed with the use of an UAS, the average stone-free rate would be 0.753, i.e., as much as 75 % of the population would be stone free. This difference of 25 %, however, representing the average treatment effect, was not significant (ATE: 0.248; *P* = 0.604). Using the IPWRA analysis on only the treated population in the estimations revealed no significant difference between using and not using of a UAS (31 %; ATET: 0.311; *P* = 0.523).

**Table 4:** Results of the inverse-probability-weighted regression adjustment

Stone-free status	Coefficient	SE	*P* value	95 % CI
Potential outcome means (POM)				
AccSh				
No (0) *A*	0.504	0.478	0.291	−0.432, 1.441
Yes (0) *B*	0.753	0.012	0.000	0.730, 0.776
Outcome model (0) for the non-treated				
Operating time	0.000	0.000	0.620	0.000, 0.000
Intra-operative complications	−0.306	2.468	0.901	−5.143, 4.530
Postoperative complications	0.845	1.483	0.569	−2.062, 3.751
Constant	−0.035	2.088	0.986	−4.127, 4.056
Outcome model (1) for the treated				
Operating time	0.000	0.000	0.119	0.000, 0.000
Intra-operative complications	−0.237	0.842	0.779	−1.887, 1.414
Postoperative complications	−0.957	0.867	0.270	−2.656, 0.742
Constant	1.194	0.069	0.000	1.058, 1.329
Treatment model				
Stone burden	0.000	0.001	0.999	−0.002, 0.002
Stone location	0.099	0.039	0.012	0.022, 0.176
Stone size	0.054	0.028	0.059	−0.002, 0.110
Previous treatment	0.201	0.112	0.074	−0.019, 0.420
Academic center	−0.252	0.112	0.024	−0.472, −0.033
Case volume	−0.002	0.000	0.000	−0.002, −0.001
Constant	0.370	0.227	0.103	−0.075, 0.815

## Discussion

Flexible URS is currently recommended by the European Association of Urology Urolithiasis Guidelines for the treatment of renal stones sized up to 1.5 cm [10]. Of note, currently most urologists recommend ‘dusting’ kidney stones when using flexible URS rather than fragmentation and removal of fragments, especially when treating larger sized stones. Consequently, one may question differences in outcome of SFR depending on the application of fragmentation or dusting of the stone. Whereas in smaller sized stones, depending on the localization and composition either dusting or fragmentation is used, in larger sized stones often the use of dusting followed by fragmentation is applied. At the very end, the SFR is based on the fragments left following fragmentation. An UAS can be most instrumental to remove the multitude of fragments.

Reports have been made that the use of the UAS impacts on SFRs following flexible URS. L’Esperance et al. [1] conducted a retrospective review of 256 ureteroscopy procedures for the removal of renal calculi performed between 1997 and 2003 (173 with UAS and 83 without). The groups were similar in age, sex and stone burden. Overall SFRs were 79 and 67 % in the UAS group and non-UAS group, respectively (*P* = 0.042). In contrast, reports from Berquet et al. [11] (86 % in UAS group vs. 87 % in non-UAS group; *n* = 280) and Kourambas et al. [2] (78 % in the UAS groups vs. 85 % in non-UAS group; *n* = 59) reported no difference in SFRs with or without a UAS. In the current study with large patient population, SFR was 73.9 % in the UAS group versus 82.8 % in the non-UAS group. This indicates an UAS is not primarily used to increase SFR. However, there were a number of limitations to the study. One limitation is that the reason to use an UAS, such as fragments removal, facilitate access or decrease intra-renal pressure, was not recorded, and consequently, the primary reason for its use was unknown. Besides possible added efficacy of the use of an UAS, also the added safety of the use of an UAS without increased risk of ureteral damage is important. From Table 2 in the present work one can conclude that UAS usage did not increase the risk of ureteral damage (perforation 1.1 vs. 1.2 %) nor bleeding (2.1 vs. 1.2 %), whereas Table 3 shows the reduced incidence of postoperative infectious complications (fever, UTI, sepsis 28.6, 18.6 and 4.3 % vs. 39.1, 23.9 and 15.2 % for the UAS vs. the non-UAS group accordingly). Noteworthy is that several UAS are marketed involving different lengths, sizes and characteristics, which relate to ureter status and anatomy. A study on compatibility of the different UASs with flexible URS showed the 12/14 F UAS to be a “universal” UAS that accepts the currently available flexible ureteroscopes [12]. But the smaller sized ureteroscopes have fueled the development of smaller sized UAS; at present 10/12 and 11/13 are predominantly used [12], resulting in less complications.

Another limitation may be the evaluation of SFR. We are aware that standardization of evaluation of stone management is often lacking. This has been highlighted in a recent communication on standardization on terminology following PCNL [13]. Ideally, a CT scan can best evaluate SFR following ureteroscopy. In the present real-life work, the SFR was evaluated by CT in a limited number of cases, whereas otherwise plain abdominal X-ray, renal ultrasound or intra-operative findings were used.

Finally, a more obvious limitation is the lack of randomization. As the subject characteristics between treatment groups (UAS or non-UAS) were imbalanced, the direct comparison of treatment groups was inappropriate. To overcome this, the IPWRA method [14] was used to determine whether the use an UAS impacted on SFR and correcting this for intra- and postoperative characteristics. The results showed no significant difference in SFR between the using and not using of a UAS.

In conclusion, in the URS Global Study there was no difference in SFR when a UAS was used or not. This result was independent from baseline characteristics and corrected for possible intra- and postoperative confounders. Intra-operative complication rates were favorable for the UAS group, including less ureteral damage or bleeding.

## References

[1] L’Esperance JO, Ekeruo WO, Scales CD, Marguet CG, Springhart WP, Maloney ME (2005). Effect of ureteral access sheath on stone-free rates in patients undergoing ureteroscopic management of renal calculi. Urology.

[2] Kourambas J, Byrne RR, Preminger GM (2001). Does a ureteral access sheath facilitate ureteroscopy?. J Urol.

[3] Stern JM, Yiee J, Park S (2007). Safety and efficacy of ureteral access sheaths. J Endourol.

[4] Rehman J, Monga M, Landman J, Lee DI, Felfela T, Conradie MC (2003). Characterization of intrapelvic pressure during ureteropyeloscopy with ureteral access sheaths. Urology.

[5] Auge BK, Pietrow PK, Lallas CD (2004). Ureteral access sheath provides protection against elevated renal pressures during routine flexible ureteroscopic stone manipulation. J Endourol.

[6] Lallas CD, Auge BK, Raj GV, Santa-Cruz R, Madden JF, Preminger GM (2002). Laser Doppler flowmetric determination of ureteral blood flow after ureteral access sheath placement. J Endourol.

[7] Traxer O, Thomas A (2013). Prospective evaluation and classification of ureteral wall injuries resulting from insertion of a ureteral access sheath during retrograde intrarenal surgery. J Urol.

[8] De la Rosette J, Denstedt J, Geavlete P, Keeley F, Matsuda T, Pearle M (2014). The clinical research office of the endourological society ureteroscopy global study: indications, complications, and outcomes in 11,885 patients. J Endourol.

[9] Xie J, Liu C (2005). Adjusted Kaplan–Meier estimator and log-rank test with inverse probability of treatment weighting for survival data. Stat Med.

[10] Turk C, Knoll T, Petrik A, Sarica K, Skolarikos A, Straub M et al (2014) Guidelines on urolithiasis. European Association of Urology. http://www.uroweb.org/gls/pdf/22%20Urolithiasis_LR.pdf. Accessed 9 Jan 2015

[11] Berquet G, Prunel P, Verhoest G, Mathieu R, Bensalah K (2014). The use of ureteral access sheath does not improve stone-free rate after ureteroscopy for upper urinary tract stones. World J Urol.

[12] Al-Qahtani SM, Letendre J, Thomas A, Natalin R, Saussez T, Traxer O (2014). Which ureteral access sheath is compatible with your flexible ureteroscope?. J Endourol.

[13] Opondo D, Gravas S, Joyce A, Pearle M, Matsuda T, Sun Y, Assimos D, Denstedt J, de la Rosette J (2014). Standardization of patient outcomes reporting in percutaneous nephrolithotomy. J Endourol.

[14] Sugihara M (2010). Survival analysis using inverse probability of treatment weighted methods based on the generalized propensity score. Pharm Stat.

